# Research progress and prospects of benefit-risk assessment methods for umbilical cord mesenchymal stem cell transplantation in the clinical treatment of spinal cord injury

**DOI:** 10.1186/s13287-024-03797-y

**Published:** 2024-07-02

**Authors:** Ruoqi Shen, Yubao Lu, Chaoyang Cai, Ziming Wang, Jiayu Zhao, Yingjie Wu, Yinian Zhang, Yang Yang

**Affiliations:** 1 Department of Spine Surgery, The Third Affiliated Hospital of Sun Yat-Sen University, No. 600 Tianhe Road, Tianhe District, Guangzhou, Guangdong Province China; 2 National Medical Products Administration (NMPA) Key Laboratory for Quality Research and Evaluation of Cell Products, No. 600 Tianhe Road, Tianhe District, Guangzhou, Guangdong Province China; 3 Guangdong Provincial Center for Engineering and Technology Research of Minimally Invasive Spine Surgery, No. 600 Tianhe Road, Tianhe District, Guangzhou, Guangdong Province China; 4 Guangdong Provincial Center for Quality Control of Minimally Invasive Spine Surgery, No. 600 Tianhe Road, Tianhe District, Guangzhou, Guangdong Province China; 5 Department of Neuro-Oncological Surgery, Neurosurgery Center, Zhujiang Hospital of Southern Medical University, Guangzhou, China

**Keywords:** Spinal cord injury, Umbilical cord mesenchymal stem cells, Therapeutic strategies, Benefit-risk assessment, Functional evaluation methods

## Abstract

Over the past decade, we have witnessed the development of cell transplantation as a new strategy for repairing spinal cord injury (SCI). However, due to the complexity of the central nervous system (CNS), achieving successful clinical translation remains a significant challenge. Human umbilical cord mesenchymal stem cells (hUMSCs) possess distinct advantages, such as easy collection, lack of ethical concerns, high self-renewal ability, multilineage differentiation potential, and immunomodulatory properties. hUMSCs are promising for regenerating the injured spinal cord to a significant extent. At the same time, for advancing SCI treatment, the appropriate benefit and risk evaluation methods play a pivotal role in determining the clinical applicability of treatment plans. Hence, this study discusses the advantages and risks of hUMSCs in SCI treatment across four dimensions—comprehensive evaluation of motor and sensory function, imaging, electrophysiology, and autonomic nervous system (ANS) function—aiming to improve the rationality of relevant clinical research and the feasibility of clinical translation.

## Background

Spinal cord injury (SCI) is a serious disease of the central nervous system (CNS) that often leads to not only the loss of motor and sensory functions but also autonomic nervous system (ANS) dysfunction below the level of injury (including the loss of function or dysfunction of the cardiovascular system, digestive system, urinary system and endocrine system). SCI has therefore been considered a major medical challenge that needs to be addressed [1, 2]. Due to their loss of working ability and ability to take care of themselves, SCI patients often experience heavy social and family burdens, which come from not only high medical expenses but also the complete loss of the patient's social participation ability and the human cost of care. According to incomplete statistics, an SCI patient with a guaranteed quality of life incurs up to $5,655,557 in annual healthcare-related costs, and the costs increase with age. Importantly, this estimate does not include the immeasurable social losses caused by the inability of patients and caregivers to participate in social activities [3]. However, no treatment has been clinically shown to be effective in curing SCI, so finding a treatment that can stabilize and effectively improve the neurological function of SCI patients is a key medical and societal concern.

The complete loss of function of other organs, such as the heart, liver, and skin, can be reversed by transplantation [4, 5]. However, it seems that the CNS is still an "absolute forbidden area" for organ transplantation at present; due to this specificity, there is no hope for the restoration of neurological function after SCI through the transplantation of a donor spinal cord at present or even in the next few decades. Cell transplantation is a partial direct solution to this dilemma [6]. At present, a variety of cells, including embryonic stem cells, induced pluripotent stem cells, neural stem cells, neural progenitor cells, mesenchymal stem cells, oligodendrocyte progenitor cells, olfactory ensheathing cells, and Schwann cells, have been shown to promote neural function recovery after transplantation in SCI, and many clinical studies have involved preclinical exploration of cell transplantation in SCI [7]. Among them, stem cells seem to be the most clinically translatable cell choice for transplantation, but as of now, there is still a long way to go before stem cell transplantation can be introduced into the clinical treatment of SCI [8]. With their unique advantages and complete preparation system, human umbilical cord mesenchymal stem cells (hUMSCs) have become the most promising cell type for SCI treatment [9]. Therefore, in this paper, the urgent problems that need to be solved in the clinical translation of stem cell transplantation in SCI are reviewed, aiming to provide feasible and improved solutions for the development of stem cell transplantation therapy for SCI.

## Therapeutic role of hUMSCs

In cell transplantation therapy, there are strict requirements for the therapeutic efficacy of cells; the self-renewal ability of cells; and the feasibility of processes such as acquisition, preparation, transportation and preservation. hUMSCs exhibit excellent characteristics in the above aspects. First, as a kind of stem cell, hUMSCs have good self-renewal and multidirectional differentiation potential, so hUMSCs can continuously proliferate and differentiate into one or more cell types under specific conditions and participate in the repair and reconstruction of human tissues and organs for therapeutic purposes [10]. Moreover, hUMSCs can be easily harvested, isolated, cultured, amplified, and purified; furthermore, after multiple passages and expansions, the viability and therapeutic function of hUMSCs can be maintained during the initial generation [11]. In addition, the surface antigen of hUMSCs is not prominent, rejection reactions to transplanted cells are not obvious, and the matching requirements are not strict, making them easy to use in allogeneic transplantation [12, 13].

**Fig. 1:**
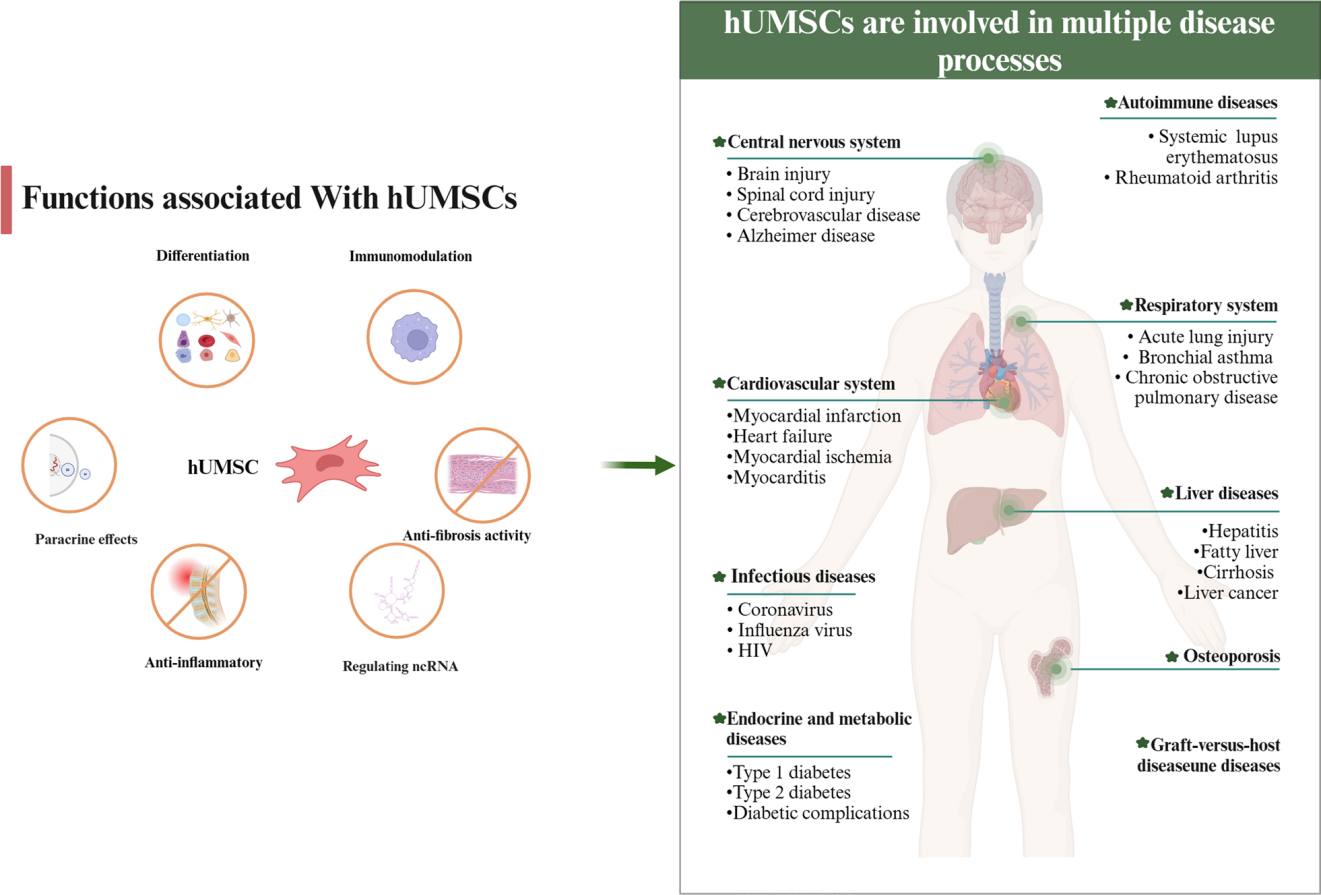
Regulatory functions and clinical applications of hUMSCs. hUMSCs possess functions including differentiation, immunomodulation, anti-inflammation, anti-fibrosis, paracrine activity, and regulation of noncoding RNA. hUMSCs are involved in the treatment of endocrine and metabolic diseases, liver diseases, autoimmune diseases, CNS disorders, cardiovascular diseases, respiratory system diseases, infectious diseases, graft-versus-host disease, musculoskeletal diseases, etc.

Currently, hUMSCs are used to treat various diseases. In Fig. 1, we provide a detailed illustration of the various diseases treated by hUMSCs, highlighting the diverse therapeutic roles of these cells across multiple disease categories. These cells have several different therapeutic roles that are essential for their translation into clinical applications [1]. Differentiation: The differentiation induced by hUMSCs can promote tissue regeneration and, on this basis, play a role in the reconstruction of damaged tissue functions [2, 14]. Immunomodulation: hUMSCs can inhibit the proliferation of immune cells such as T cells, B cells, and follicular helper T (Tfh) cells; induce the differentiation of macrophages from a proinflammatory phenotype to an anti-inflammatory phenotype; and attenuate inflammation by secreting IL-10 and IL-4 [15]. The regulation of the above immune response can effectively reduce the extent and severity of tissue damage and simultaneously effectively promote tissue repair [3]. Anti-inflammatory effect: hUMSCs can reduce inflammation and oxidative stress by inhibiting the secretion of IL-1β, IL-8, and tumour necrosis factor-α (TNF-α), thereby further reducing the degree of cellular damage and inhibiting apoptosis [4, 16–19]. Antifibrotic activity: hUMSCs stimulate the apoptosis of fibrosis-related cells and the secretion of cytokines with antifibrotic functions to reduce the degree of tissue fibrosis. At the same time, the antifibrotic effect of hUMSCs can also be achieved by regulating relevant signalling pathways and promoting vascular remodelling [5, 20–24]. Paracrine effects: hUMSCs can secrete soluble molecules such as keratinocyte growth factor (KGF), fibroblast growth factor (FGF), hepatocyte growth factor (HGF), vascular endothelial growth factor (VEGF), epidermal growth factor (EGF) and other cytokines to promote tissue regeneration [6, 25–28]. Regulating the expression of noncoding RNA (ncRNA): hUMSCs can affect the localized expression of ncRNA, including microRNAs (miRNAs), long noncoding RNAs (lncRNAs) and circular RNAs (circRNAs), in damaged tissue and indirectly regulate their target genes for therapeutic purposes [16, 29, 30].

Currently, hUMSCs are used to treat more than a dozen diseases [12], including endocrine and metabolic diseases (including but not limited to type 1 diabetes [31], type 2 diabetes [32], diabetic foot [33], diabetic nephropathy [34], diabetic cardiomyopathy [33, 35] diabetic retinopathy [36] and other complications), liver diseases (including but not limited to hepatitis [37], fatty liver [38], cirrhosis [39, 40], and liver cancer [41]), autoimmune diseases (including but not limited to systemic lupus erythematosus [42] and rheumatoid arthritis [43]), CNS diseases (including but not limited to brain injury [44], SCI [45], cerebrovascular disease [46], and Alzheimer's disease [47]), cardiovascular system diseases (including but not limited to myocardial infarction [48], heart failure [49], myocardial ischaemia [50], and myocarditis [51]), respiratory diseases (including but not limited to acute lung injury [52], bronchial asthma [53], and chronic obstructive pulmonary disease [54]), infectious diseases (including but not limited to coronavirus [55], influenza virus [56], HIV [57] and other viral infections), graft-versus-host disease [58] and osteoporosis [59], etc*.* (Fig. 1).

## Progress in the research and application of hUMSCs in SCI

Although SCI is a serious CNS condition, there are no effective pharmacological or nonpharmacological means to help SCI patients effectively repair and rebuild their destroyed neurological functions. Therefore, there is an urgent need for effective therapeutic approaches for the clinical treatment of SCI [60]. Transplantation therapy based on cells (i.e., mature cells) or stem cells (i.e., undifferentiated cells or partially differentiated cells that can differentiate and proliferate) is currently one of the most promising strategies for the treatment of SCI [61]. hUMSCs were among the first candidate cells to be tested in clinical trials. With the excellent characteristics of hUMSCs, substantial progress has been made in clinical trials of cell therapy for SCI.

In a clinical study by Hongbin Cheng et al. [62], 34 patients with thoracolumbar SCI were randomly divided into 3 groups: the hUMSC transplantation group underwent two computed tomography (CT)-guided hUMSC transplantations (amount of transplanted cells: 4 × 10^7^); the rehabilitation group received rehabilitation treatment; and the blank control group did not receive any special treatment. In this study, the American Spinal Cord Injury Association Impairment Scale (AIS), American Spinal Injury Association Impairment Scale A (ASIA), manual muscle strength and muscle tension scale, and Barthel index were used to evaluate clinical efficacy. The results showed that 7 out of 10 patients in the hUMSC group experienced significant and stable improvements in motion, self-care ability and muscle tension; 5 out of 14 patients (36%) in the rehabilitation group also experienced certain improvements in these aspects. The urodynamic examination results showed that the maximum urinary flow rate and maximum bladder capacity of the patients in the hUMSC group increased, while the residual urine volume (RUV) and maximum detrusor pressure decreased. The maximum bladder capacity of the rehabilitation group decreased, but the maximum urinary flow rate, RUV and maximum detrusor pressure did not change significantly. Therefore, this study proved that hUMSCs can effectively promote the recovery of motor function and urinary function in SCI patients, and no adverse reactions were found at a transplantation volume of 4 × 10^7^. In another randomized, double-blind, crossover, placebo-controlled phase 1/2a clinical trial (NCT03003364) [63], researchers randomly assigned 10 patients with chronic complete SCI (including 7 male SCI patients and 3 female SCI patients aged 25–47 years, all with chronic complete SCI) to receive an hUMSC (1 × 10^7^) transplant or placebo treatment. The researchers conducted clinical assessments (AIS score, spasticity, neuropathic pain, electrical perception and pain threshold evaluations); measured lower limb motor evoked potentials (MEPs) and sensory evoked potentials (SEPs); and administered the Spinal Cord Independence Measure and World Health Organization Quality of Life Brief Version questionnaire at baseline and 1, 3 and 6 months after the intervention. Urodynamic examination, urine-specific quality of life evaluation (Qualiveen questionnaire), anorectal manometry, functional assessment of bowel dysfunction (Rome III diagnostic questionnaire) and assessment of the severity of faecal incontinence (Wexner score) were conducted at baseline and 6 months after the intervention. This study revealed that after the transplantation of hUMSCs into patients with SCI, the pinprick sensation of patients in the hUMSC treatment group significantly increased compared with that of patients in the control group. Other clinically relevant indicators, such as increases in bladder maximum capacity and compliance and decreases in bladder neurogenic hyperactivity and external sphincter dyssynergy, differed only within individual patients. However, no differences were observed in motor function, spasticity, MEPs, SEPs, bowel function, quality of life, or independence measures between the hUMSC treatment group and the control group in this study. Therefore, this study also demonstrated that intrathecal transplantation of hUMSCs is a safe intervention method for SCI patients and that a single intrathecal injection of hUMSCs can increase the sensation of adjacent injured segments in patients with chronic complete SCI. In a prospective, single-arm, single-centre study conducted by Yang Yang et al. [9], 102 subjects were evaluated for safety, and 41 subjects were evaluated for efficacy. Multiple hUMSC transplantations were performed for patients with SCI in this study. The transplantation dose was 1 × 10^6^ kg/time. A total of four transplantations were performed during the study period, with one month between each transplantation. The researchers followed up with the patients 1, 3, 6, and 12 months after the last dose and compared the ASIA score and the SCI Functional Rating Scale score of the International Association of Neurorestoratology (IANR-SCIFRS) score as the main observation indexes; pinprick sensation, light touch, motor and sphincter function, muscle spasticity and spasm, autonomic system, bladder and bowel function, RUV and magnetic resonance imaging (MRI) findings were used as secondary observation indices to comprehensively evaluate the patients’ neurological function. In terms of safety, SCI patients experience mild adverse effects after undergoing hUMSC transplantation, including fever (14.1%), headache (4.2%), a transient increase in muscle tone (1.6%) and dizziness (1.3%), but these adverse reactions could be completely eliminated by conservative treatment. In terms of effectiveness, the total ASIA and IANR-SCIFRS scores significantly increased compared with the baseline at different time points during the study, and these improvements were mainly reflected in improvements in pinprick sensation, light touch, motor function, and sphincter function. Additionally, the subjects experienced a sustained and significant decrease in muscle spasms. Regarding muscle spasms and ANS, bladder and bowel function, RUV and MRI data/imaging at the final follow-up showed significant improvement compared to the first collection. Moreover, hUMSC transplantation improved the recovery of neurological function in patients with SCI regardless of the level, severity and chronicity of the injury (Table 1).

**Table 1:** Multinational study assessing treatment efficacy of hUMSCs on spinal cord injury in patients with multidimensional assessments

Study	Total participants	Phase	Assessment time	Country	ASIA	Muscle spasticity and spasm	Barthel Index	Neuropathic pain	Electrical perception and pain thresholds	MEPs and SEPs	WHOQOL-BREF	IANR-SCIFRs	Urodynamic examination	Anorectal manometry	Wexner score	Pinprick sensation	Light touch score	Motor and sphincter function	Autonomic system	Bladder and bowel function	MRI
Hongbin Cheng et al. [62]	34	Chronic	6 months before and after therapy	China	√	√	√						√								
Albu et al. [63]	10 (7 M/3 F)	Chronic	1, 3, and 6 months before and after therapy	Spain	√	√		√	√	√	√	√	√	√	√			√		√	
Yang yang et al. [9]	143	Chronic	1, 3, 6, and 12 months after therapy	China	√	√					√	√	√		√	√	√	√	√	√	√

In addition to simply transplanting hUMSCs, loading hUMSCs on supportive tissue engineering scaffolds before they are implanted into patients is a treatment option with clinical application potential. Two clinical studies published by Zhifeng Xiao [64] and Wusheng Deng [65] demonstrated that collagen scaffold-loaded hUMSCs can effectively restore intestinal and bladder functions after transplantation into SCI patients and significantly improve sensory, motor and self-care abilities, while ASIA scores and daily living ability scores also improved significantly. In addition, cotransplanting hUMSCs with other cells could be effective at enhancing the therapeutic efficacy of hUMSCs. Multiple studies have shown that the combination of hUMSCs with human neural stem cells (HNSCs), glial cell line-derived neurotrophic factor (GDNF) or hypoxia can improve the outcomes of cell therapy [66–68], but unfortunately, the efficacies of these combination regimens have not been verified in clinical trials; therefore, further clinical trials are needed to prove the therapeutic efficacy of the above combination treatment regimens in the treatment of patients with SCI.

It can be seen that hUMSCs have a certain degree of efficacy in the treatment of SCI, but it is not enough to free patients from the pain caused by SCI. Therefore, a more comprehensive treatment program is needed to further increase the benefits of hUMSCs in the treatment of SCI. Although the use of biomaterial-loaded hUMSCs and multicellular cotransplantation has improved the efficacy of hUMSCs in treating SCI to a certain extent, unfortunately, the efficacy of these options has not been validated in clinical studies. Therefore, more clinical studies are needed to validate the efficacy of these combined treatment regimens. In addition, most of the recent clinical studies of hUMSCs for SCI are single-centre studies, so we suggest that multicentre combination studies be conducted when available to improve the quality of evidence in clinical studies.

## Risk–benefit evaluation of hUMSCs for SCI treatment

For the development of SCI treatment plans, adopting appropriate risk–benefit evaluation methods is important for evaluating whether the treatment plan is clinically applicable. Therefore, we investigated methods that can be used to assess the benefit and risk of receiving hUMSCs for SCI treatment with the aim of improving the rationality of relevant clinical research and the feasibility of clinical translation.

### Comprehensive evaluation method

For the assessment of the degree of injury and treatment effect in SCI patients, comprehensive evaluation from multiple perspectives is an important method with unified standards. The methods that can be used for the comprehensive evaluation of SCI mainly include the AIS, International Standards for Neurological Classification of SCI (ISNCSCI) and IANR-SCIFRS. Currently, among the tools used to predict prognosis after SCI, the ISNCSCI classification criteria and AIS are the most commonly used tools [69]. The ISNCSCI classification criteria were developed to standardize examination techniques and provide consistent terminology and definitions within the SCI classification system to detect changes in neurological function over time. In the first edition, published by Asia in 1982, the Frankel scale was used to classify injury severity [70]. Major revisions adopted in 1992 included replacing the Frankel scale with the AIS and using sacrum-sparing criteria to define the completeness of injury. Prior to 1992, complete impairment was defined as sensory and motor deficits greater than three levels below the neurological level of injury (NLI). Since 1992, the determination of function integrity has relied on sensory or motor function at the lowest sacral level (sensory function at the S4-S5 dermatome and presence of deep anal pressure (DAP) or voluntary anal contraction (VAC)). The originally reported sacrum-sparing definition was a more s classification, with fewer patients transitioning from an incomplete to a complete injury state, which was confirmed in a more recent analysis [71, 72]. The European Multicenter Study about Spinal Cord Injury (EMSCI), National Spinal Cord Injury Model Systems (SCIMS), Rick Hansen Spinal Cord Registry Study The Injury Registry (RHSCIR) and North American Clinical Trials Network (NACTN) have played important roles in the collection of neurological data from thousands of patients with traumatic SCI and have been used to characterize neurological recovery after SCI [73, 74].

The treatment of SCI treatment through hUMSC transplantation has attracted widespread attention, and the effectiveness and safety of this treatment are being validated and studied through clinical trials in several countries [9, 62–65, 107]. In evaluating the effects of rehabilitation after SCI, most studies have used the ASIA score as the primary rating criterion, with MEPs and SEPs and urodynamics as secondary evaluation indices (Table 2). However, the methods to assess all of these indices are to some extent subjective to the assessment operator and therefore need to be carried out by more than one person in a well-trained and strictly blinded manner; otherwise, the conclusions of clinical studies will be biased. Therefore, it is necessary to conduct strict training and qualification assessments for evaluators. At the same time, with the continuous development of artificial intelligence (AI) technology, which provides the possibility of homogenized and unbiased assessment of patients in different centres, it may be of great prospective significance to develop corresponding algorithms to assist individuals in clinical research and practice in treatment efficacy assessment.

**Table 2:** Inventory of published clinical studies

Author	Registration number	Country	Total participant	Phase	Dose	ASIA	MEPs and SEPs	Urodynamic	Reference number
Xiao et al.	NCT02510365	China	2 M	Acute	4 × 10^7^	√	√		[64]
Cheng et al.	NCT01393977	China	34	Chronic	4 × 10^7^	√		√	[62]
Albu et al.	NCT03003364	Spain	10 (7 M/3 F)	Chronic	1 × 10^7^	√	√	√	[63]
Yang et al.	NCT02481440	China	143	Chronic	1 × 10^6^	√		√	[9]
Deng et al.	NCT 02510365	China	40 (12/28)	Acute	4 × 10^7^	√	√	√	[65]
Zhao et al.	NCT02352077	China	8 (7 M/1F)	Chronic	4 × 10^7^	√	√		[107]

### Imaging assessment

Imaging examination is currently an important source of evidence in clinical medicine. Therefore, for patients with SCI, appropriate imaging examinations can be used to evaluate the condition in a timely manner.

**Fig. 2:**
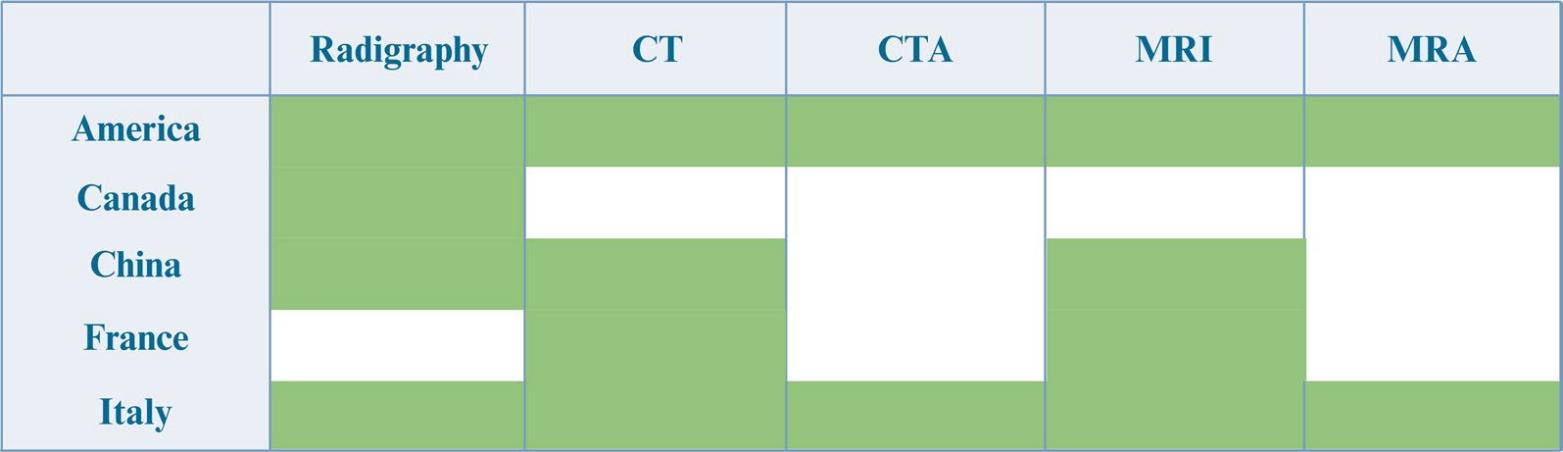
Comparative imaging guidelines for spinal cord injury across countries. The chart represents the imaging modalities recommended in the guidelines of different countries

Currently, X-ray, CT and MRI are the most commonly used imaging methods for SCI diagnosis and condition evaluation. However, X-ray and CT involve a certain degree of radiation exposure, so it is necessary to consider whether the examination itself will cause unnecessary radiation hazards to patients. To address this problem, numerous clinical guidelines have been developed that can be used to assess the need for imaging in patients. Figure 2 presents several key guidelines, including the New Orleans Criteria (NOC) [75], the expert consensus on the diagnosis and treatment of traumatic SCI, French recommendations for the management of patients with SCI or at risk of SCI [76], imaging of cervical spine traumas [77], the Canadian CT Spine Rule (CCTSR) [78], and the National Emergency X-Radiography Utilization Study (NEXUS) [79, 80]. In addition, the American College of Radiology has issued relevant guidelines for the selection of appropriate imaging examinations in various clinical situations to make hierarchical recommendations. This guideline explains the radiation exposure in X-ray and CT, which can provide comprehensive guidance for clinicians in the choice of imaging examinations [81, 82] (Fig. 2).

According to the American College of Radiology standards, for patients with suspected SCI, CT is the most appropriate initial examination for patients with mild, moderate, or severe trauma if it is not contraindicated by NOC, CCTSR, or Nexus standards [81]. However, X-ray is not suitable for any patient with acute SCI. If vascular injury is suspected, CT angiography (CTA) is recommended, and MRI and magnetic resonance angiography (MRA) are also considered suitable for initial examination in SCI patients with suspected vascular injury. Otherwise, in any other case, MRI is not considered an appropriate initial imaging test for SCI [81]. After a patient is diagnosed with SCI, as the patient's neurological status changes, regular use of CT is an effective way to assess the patient’s condition throughout the clinical course, but when CT findings cannot explain new symptoms, MRI examination is needed to clarify the progression of the injury. Notably, CT examinations are suitable only for regular follow-up with long intervals. Repeated CT examinations in the short term are not recommended unless necessary, as this may expose patients to excessive levels of radiation [81]. In one study, of 367 patients with SCI diagnosed by MRI, only one had a negative CT scan after trauma, yielding a false-negative rate of 0.3% [83]. However, CT imaging of the spinal cord is not ideal, so any patient with suspected SCI should be evaluated with MRI because MRI can provide information about not only the location and severity of the injury in the spine but also the cause of the injury, which may include haematoma, bone fragments, or disc herniation [84]. Therefore, for SCIs, the gold standard in the diagnostic stage is CT, which means that when SCI occurs, a CT examination should be performed first to check for bone damage. After damage to the spinal structure is confirmed, MRI is recommended for further confirmation. When vascular injury is suspected, CTA or MRA can be selected for examination [82]. Compared with MRI, the advantages of CT include wide availability, fast speed, low cost, and no need for metal screening; CT has extremely high sensitivity for detecting spinal fractures, and its sensitivity for detecting bleeding is close to that of MRI. Compared with CT, the advantages of MRI are that it has better spinal cord imaging quality, there is no radiation risk, and repeated examinations can be performed in a short period to achieve continuous monitoring of changes in a patient’s condition [85–87].

From the perspective of accessibility and diagnostic efficacy, the use of CT and MRI for diagnostic imaging and evaluation of patients with SCI is recommended, while X-ray can be an important adjunct method for primary care providers who do not have access to the equipment for these methods. Regardless of the method used, there is no doubt about the importance of imaging in the diagnosis and management of SCI.

### Electrophysiological assessment

Imaging can reveal nerve damage and recovery in SCI patients at the tissue structure level. However, there is often no fixed correspondence between the neurological function of SCI patients and the degree of damage to the spinal cord structure, so functional examinations are needed to provide a more objective and comprehensive assessment of spinal cord function. Electrophysiological assessment can accurately assess the ability of the spinal cord to conduct and process nerve signals and therefore can increase the therapeutic efficacy of clinicians' intervention methods (such as hUMSC transplantation) in aspects such as neuroplasticity, axonal growth, and remyelination [88–90]. Therefore, over the past few decades, electrophysiological examination has gradually become recognized as an important examination method for diagnosing and evaluating the condition of patients with SCI, predicting functional outcomes, planning therapeutic intervention plans, and evaluating treatment efficacy [91]. The somatosensory evoked potential (SSEP), cranial electrotherapy stimulation (CHEP), LEP and MEP are used to test the nerve signal transmission function of the spinal cord, including the uploading of somatosensory signals and the transmission of motor signals. Among them, SSEPs are mainly used to test the dorsal column function of the spinal cord, while CHEPs or laser-evoked potentials (LEPs) are mainly used to test the spinothalamic pathway [92]. The detection of SSEPs confirms the synchronous signalling of large-diameter myelinated fibres within the peripheral and ascending dorsal columns of the spinal cord, whereas CHEPs and LEPs reflect the signalling of small-diameter afferent pathways [93]. MEP is mainly used to test the downwards conduction of motor signals, so transcranial stimulation of the motor cortex to induce this conduction. Transcranial stimulation relies on the signal transduction of direct and indirect pathways. The direct pathway mainly refers to the corticospinal tract, and the indirect pathway mainly includes the reticulospinal and intrinsic spinal pathways [94, 95]. MEP detection can well reflect the ability of the CNS to control peripheral target organs (muscles) in SCI patients through signal intensity and latency, thereby providing relevant information about the impairment of CNS function [96]. In SCI, in addition to the somatic nervous system, the ANS can also be topographically assessed through electrophysiological testing, but this assessment is limited to the sympathetic nervous system, in which sympathetic skin responses (SSRs) can help to determine the location of the lesion level and the dysfunction of the sympathetic mediolateral cell columns of the spinal cord [97]. At the same time, SSRs can also be used to assess the prognosis of potentially serious complications, such as autonomic dysreflexia [98].

Electrophysiological testing can relatively objectively assess the ability of the spinal cord to transmit neural signals and is an important means of evaluating CNS disorders. Therefore, for patients with SCIs, regular electrophysiological testing can provide important evidence for clinical efficacy assessment. At the same time, to a certain extent, electrophysiological testing results are related to the results of the comprehensive assessment, and the verification of this relationship can ensure the scientific validity and accuracy of the conclusions of clinical trials.

### Assessment of ANS function

Various neurological disorders, such as cardiovascular system dysfunction, urination disorder, defecation disorder and loss of sexual function, are important causes of the poor quality of life of SCI patients, which not only negatively affects the patient's health and well-being but also results in high nursing costs for the patient's family and society.

Blood pressure management and cardiac function testing are important factors that cannot be ignored in the follow-up and management of SCI patients. These methods mainly include monitoring tachycardia, hypotension, orthostatic hypotension, cardiovascular autonomic nerve reflex abnormalities, and blood pressure instability [99]. Therefore, regular measurements of blood pressure and heart rate in SCI patients and the creation of detailed records are important parts of the course management and follow-up of SCI patients [100].

Urinary function, defecation function, and sexual function are important factors that affect the quality of life of SCI patients. Therefore, the evaluation of these three ANS functions is important and cannot be ignored in the follow-up of SCI patients. For the evaluation of urinary function, the evaluation of urodynamic indicators, including the maximum urinary flow rate, maximum bladder capacity, RUV, and maximum detrusor pressure, is necessary [101]. At the same time, the Qualiveen questionnaire can also be a powerful tool for evaluating the quality of life associated with voiding [102]. However, traditional urodynamic testing requires invasive procedures for patients, and there is a risk of urethral injury and urinary tract infection during the insertion, indwelling and removal of urinary catheters. Therefore, less invasive methods are urgently needed to help patients undergo effective urodynamic assessments. Morteza Zakeri Nasrabadi et al*.* developed a wearable urodynamic testing device that can reduce patient pain while ensuring the reliability of the assessment [103]. Anorectal manometry is a commonly used clinical assessment method for evaluating defecation function. The Rome III diagnostic questionnaire and the Wexner score can also be used to evaluate patients' defecation function [63, 104, 105]. The assessment of sexual function mainly includes the assessment of sexual desire, sexual arousal, orgasm, ejaculation and fertility [106]. However, there is currently no effective assessment model that can be used to assess the sexual function of SCI patients, so there is an urgent need to develop an assessment scale with high reliability and usability for the assessment of quality of life in SCI palliative care.

## Conclusions

In recent decades, with the continuous societal and economic developments, SCI has gradually become a major medical problem that cannot be ignored. Although researchers worldwide have invested considerable resources and effort to solve this problem, as far as the current situation is concerned, there is still no treatment method for SCI that can enable patients to be completely cured and return to normal life and social activities. Based on the currently available clinical experimental data, hUMSCs can effectively promote the neurological recovery of patients with SCI due to the excellent safety and therapeutic efficacy of these cells. However, the current diagnosis and treatment scheme is not enough to completely rebuild the severely damaged nerve function of SCI patients, so more relevant studies are needed to further optimize this technique. An important basis for judging whether a treatment plan can be accepted by clinical workers and patients is a strict benefit-risk assessment of the treatment plan. For the treatment of SCI, the benefit and risk assessment mainly includes four aspects: comprehensive assessment, imaging assessment, electrophysiological assessment and ANS function assessment. Comprehensive assessment helps medical staff to comprehensively define and understand the course of the patient's condition to determine the appropriate treatment program. Imaging assessments provide benefits at the structural level, while electrophysiological assessments and ANS function scores provide benefits at the neurological level. Table 2 shows that the recent clinical research on the use of hUMSCs for the treatment of SCI includes multiple comprehensive evaluation models, and the comprehensive evaluation results generated with these models are highly important for the clinical application of these models. However, the therapeutic effect of hUMSC transplantation alone is not satisfactory, so the combination of tissue engineering scaffolds, neuroelectric/magnetic stimulation, and exercise rehabilitation to form a composite therapeutic program may be an important breakthrough to overcome the therapeutic effect bottleneck.

Overall, hUMSC transplantation for the treatment of SCI is a treatment method with great potential for clinical translation, but further optimization and improvement are needed. Therefore, comprehensive, effective and reliable risk–benefit evaluations in clinical trials are important.

## Data Availability

All the data generated or analysed during this study are included in this published article.

## Abbreviations

SCI: Spinal cord injury
CNS: Central nervous system
hUMSCs: Human umbilical cord mesenchymal stem cells
KGF: Keratinocyte growth factor
FGF: Fibroblast growth factor
HGF: Hepatocyte growth factor
VEGF: Vascular endothelial growth factor
EGF: Epidermal growth factor
ncRNA: Noncoding RNA
miRNA: MicroRNA
LncRNA: Long noncoding RNA
circRNA: Circular RNA
AIS: American Spinal Cord Injury Association
ASIA: American Spinal Injury Association Impairment Scale A score
MEPs: Lower limb motor-evoked potentials
SEPs: Sensory-evoked potentials
IANR-SCIFRS: SCI Functional Rating Scale of the International Association of Neurorestoratology
RUV: Residual urine volume
CTA: CT angiography
MRI: Magnetic resonance imaging
HNSCs: Human neural stem cells
GDNF: Glial cell line-derived neurotrophic factor
ISNCSCI: International Standards for Neurological Classification of Spinal Cord Injury
NLI: Neurological level of injury
DAP: Deep anal pressure
VAC: Voluntary anal contraction
EMSCI: European Multicentre Study of Spinal Cord Injury
SCIMS: National Spinal Cord Injury Model Systems
RHSCIR: Rick Hansen Spinal Cord Registry Study Injury Registry
NACTN: North American Clinical Trials Network
CT: Computed tomography
NOC: New Orleans Criteria
CCTSR: Canadian CT Spine Rule
NEXUS: National Emergency X-Radiography Utilization Study
MRA: Magnetic resonance angiography
SSEP: Somatosensory-evoked potential
CHEP: Cranial electrotherapy stimulation
LEP: Laser-evoked potential
ANS: Autonomic nervous system
SSRs: Sympathetic skin responses
